# Positive feedback regulation of lncRNA PVT1 and HIF2α contributes to clear cell renal cell carcinoma tumorigenesis and metastasis

**DOI:** 10.1038/s41388-021-01971-7

**Published:** 2021-07-28

**Authors:** Ming-xiao Zhang, Li-zhen Zhang, Liang-min Fu, Hao-hua Yao, Lei Tan, Zi-hao Feng, Jia-ying Li, Jun Lu, Yi-hui Pan, Guan-nan Shu, Peng-ju Li, Yi-ming Tang, Zhuang-yao Liao, Jin-huan Wei, Wei Chen, Jian-ping Guo, Jun-hang Luo, Zhen-hua Chen

**Affiliations:** 1 Department of Urology, The First Affiliated Hospital of Sun Yat-sen University, Guangzhou, People’s Republic of China; 2 Department of Urology, Nanfang Hospital, Southern Medical University, Guangzhou, People’s Republic of China; 3 Institute of Precision Medicine, The First Affiliated Hospital of Sun Yat-sen University, Guangzhou, People’s Republic of China; 4 State Key Laboratory of Oncology in South China, Sun Yat-sen University Cancer Center, Guangzhou, People’s Republic of China

**Keywords:** Metastasis, Renal cell carcinoma, Prognostic markers

## Abstract

Long noncoding RNAs (lncRNAs) have been reported to exert important roles in tumors, including clear cell renal cell carcinoma (ccRCC). PVT1 is an important oncogenic lncRNA which has critical effects on onset and development of various cancers, however, the underlying mechanism of PVT1 functioning in ccRCC remains largely unknown. *VHL* deficiency-induced HIF2α accumulation is one of the major factors for ccRCC. Here, we identified the potential molecular mechanism of PVT1 in promoting ccRCC development by stabilizing HIF2α. PVT1 was significantly upregulated in ccRCC tissues and high PVT1 expression was associated with poor prognosis of ccRCC patients. Both gain-of-function and loss-of function experiments revealed that PVT1 enhanced ccRCC cells proliferation, migration, and invasion and induced tumor angiogenesis in vitro and in vivo. Mechanistically, PVT1 interacted with HIF2α protein and enhanced its stability by protecting it from ubiquitination-dependent degradation, thereby exerting its biological significance. Meanwhile, HIF2α bound to the enhancer of PVT1 to transactivate its expression. Furthermore, HIF2α specific inhibitor could repress PVT1 expression and its oncogenic functions. Therefore, our study demonstrates that the PVT1/ HIF2α positive feedback loop involves in tumorigenesis and progression of ccRCC, which may be exploited for anticancer therapy.

## Introduction

Renal cell carcinoma (RCC) is a common malignancy of the urinary system, comprising ~3% of all cancers. The morbidity of RCC is growing by 2% per year during the past two decades globally [1]. Clear cell RCC (ccRCC) is the most common pathological type of RCC, accounting for 75–80% of all RCC patients. About 20–30% of ccRCC patients are diagnosed at an advanced stage with distant organ metastasis and exhibit poor prognosis due to ineffectiveness of various treatments [2, 3]. For non-metastatic ccRCC, although early diagnosis and aggressive surgery significantly improve outcomes, the recurrence rate is still up to 20–40% after partial or radical nephrectomy [4]. Therefore, it is highly worthwhile to explore the pathogenesis of ccRCC for better treatment strategies.

Genetically, ccRCC is associated with inactivation of von Hippel-Lindau (*VHL*) tumor suppressor gene caused by the chromatin deletion together with its frequent mutation and promoter methylation [5]. pVHL, the protein encoded by *VHL*, recruits several proteins to form the VCB (VHL-elongin C-elongin B)-Cul2 E3 ubiquitin ligase and serves as the subunit to specifically recognize substrates, such as hypoxia-inducible factor α (HIFα, including HIF1α and HIF2α). Importantly, only the hydroxylated HIFα could be targeted by pVHL for ubiquitination and degradation under normoxic conditions [6, 7]. Conceivably, either inactivation of *VHL* or hypoxic conditions could abolish the interaction of pVHL and HIFα, leading to excessive accumulation of HIFα and subsequent transcriptional activation of hypoxia-responsive genes, such as VEGF, PDGF, and Cyclin D1 [8]. More importantly, although both HIF1α and HIF2α could be regulated by pVHL in a hydroxylation-dependent manner, HIF2α, but not HIF1α, plays a critical oncoprotein role in ccRCC tumorigenesis [9–12]. To this end, small inhibitors targeting HIF2α, but not HIF1α, have been developed to repress ccRCC growth and already underwent clinical trial waiting for FDA approval for ccRCC therapies [13–15]. However, except pVHL, other upstream regulation for HIF2α is not well defined.

Long non-coding RNAs (lncRNAs), which are defined as non-coding RNA transcripts longer than 200 nucleotides, play important roles in many diseases, including cancer [16]. LncRNAs are governing diverse cellular processes, ranging from cis- to trans-regulation of gene expression and from epigenetic modulation in nucleus to post-transcriptional regulation in cytoplasm [17, 18]. Among all dysregulated lncRNAs in cancers, PVT1 is particularly compelling because emerging studies have revealed that PVT1 fulfills significant oncogenic effects in the onset and progression of various cancers, including ccRCC [19–28]. Although several regulatory mechanisms of PVT1 have been reported, such as interacting with Myc and modulating the function of microRNAs and regulation of different proteins [21, 23, 29], the underlying molecular mechanism remains to be further investigated, especially in the ccRCC setting.

Here, we identify that PVT1 plays a vital role in ccRCC growth and metastasis, and high PVT1 expression is correlated with poor overall survival (OS) and progression-free survival (PFS) of ccRCC. Mechanistically, PVT1 interacts with HIF2α and impedes ubiquitin-dependent HIF2α degradation. Moreover, PVT1 is a direct transcriptional target of HIF2α. These findings together suggest that the positive feedback loop between PVT1 and HIF2α may promote tumor development and serve as a promising therapeutic target of ccRCC.

## Results

### PVT1 is upregulated in ccRCC tissues and high PVT1 level associates with poor prognosis

**Fig. 1:**
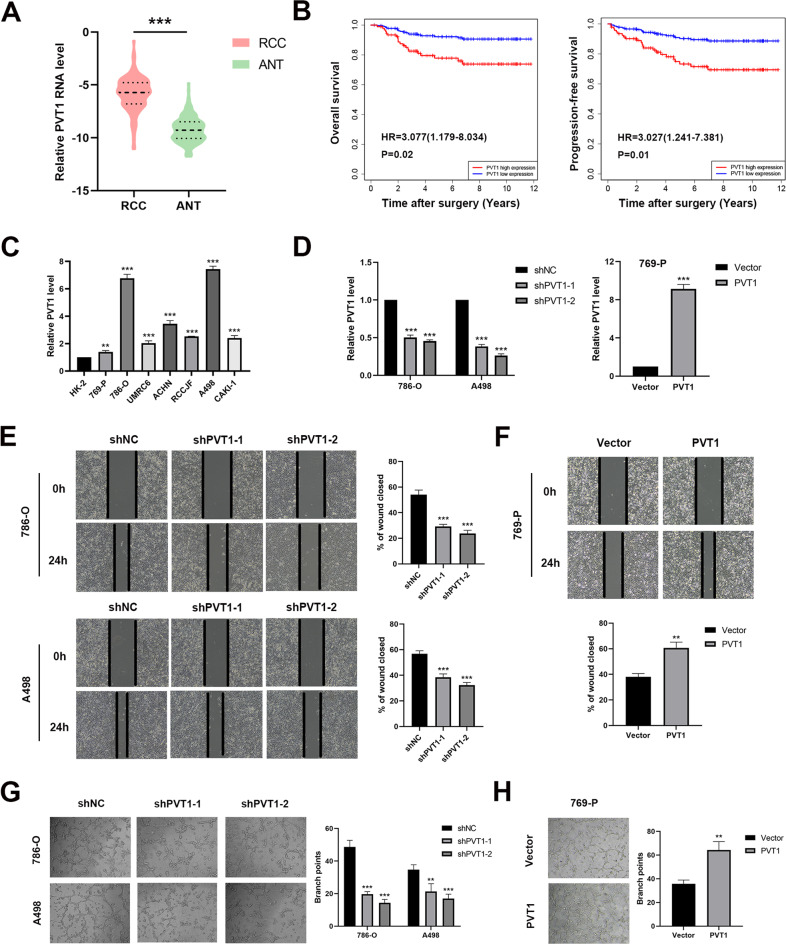
High PVT1 expression in ccRCC tissues and cells associates with poor clinical outcomes and malignant phenotypes respectively. **A** Relative PVT1 expression level was assessed by qPCR in 140 pairs of ccRCC and the matched adjacent normal tissues. **B** Survival analysis showed that high PVT1 expression was correlated with poor OS and PFS in our ccRCC cohort. **C** Expression levels of PVT1 in immortalized proximal tubule epithelial cells (HK-2) and different ccRCC cell lines were detected. **D** PVT1 was silenced in 786-O and A498 cells by two different shRNA (left panel) and overexpressed in 769-P cells (right panel). **E**, **F** Wound-healing assays showed that knockdown of PVT1 markedly impaired mobility of ccRCC cells, whereas overexpression of PVT1 enhanced it. Representative images and results of quantitative analysis were shown. Wound areas were calculated by Image J. **G**, **H** The tube formation of HUVECs was repressed when cultured in conditioned medium from PVT1-silencing cells, but promoted when cultured in conditioned medium from PVT1-overexpression cells. **P < 0.01, ***P < 0.001.

The biological significance of PVT1 was first explored using human tumor tissues and the matched adjacent normal tissues. qPCR analysis of our ccRCC cohort revealed that PVT1 expression was significantly elevated in ccRCC tissues (Fig. 1A), which was further confirmed by analysis of the transcriptome data from the TCGA KIRC cohort (Supplementary Fig. S1A). Using the median level of PVT1 as the cut-off value, we stratified the ccRCC patients into low- and high-PVT1 expression groups. The Kaplan-Meier survival analysis demonstrated that high-PVT1 expression group showed greater tumor size, increased lymph node metastasis, and distant metastasis compared with low-PVT1 expression group (Supplementary Table 1). Moreover, high PVT1 expression was also correlated with poor overall survival (OS) and progression-free survival (PFS) in our ccRCC cohort (Fig. 1B). Similar results were also observed in the analysis of TCGA KIRC cohort (Supplementary Fig. S1B). These results together suggest that PVT1 may play an important role in ccRCC tumorigenesis and progression, and serve as a valuable prognostic marker in ccRCC.

### PVT1 promotes the malignant phenotypes of ccRCC cells in vitro

We next explored the potential role of PVT1 in ccRCC development. To this end, we initially measured the expression of PVT1 in immortalized proximal tubule epithelial cells (HK-2) and a panel of ccRCC cell lines (769-P, 786-O, UMRC6, ACHN, RCCJF, A498, CAKI-1). Consistent with our clinical observation, PVT1 was upregulated in ccRCC cell lines compared with HK-2 cells (Fig. 1C). Several studies have reported the facilitating effects on cell proliferation, migration and invasion of PVT1 in ccRCC [25–28], thus we conducted gain and loss of function experiments to validate these oncogenic functions. Silencing PVT1 in 786-O and A498 cells or overexpressing PVT1 in 769-P cells (Fig. 1D), dramatically suppressed or promoted cell proliferation and colony formation, respectively (Supplementary Fig. S1C-F). We also conducted wound-healing assays and found that knockdown of PVT1 markedly impaired the mobility of 786-O and A498 cells, whereas overexpression of PVT1 improved the mobility of 769-P cells (Fig. 1E, F). In addition, transwell migration and matrigel invasion assays were further performed and validated that knockdown of PVT1 significantly inhibited migration and invasion of 786-O and A498 cells, and reverse results were observed in 769-P cells with ectopic expression of PVT1 (Supplementary Fig. S1G-J). More importantly, HUVECs, which were incubated with conditioned medium collected from PVT1-knockdown 786-O and A498 cells, formed fewer capillary tubes compared with control groups (Fig. 1G). In contrast, conditioned medium collected from PVT1-overexpression 769-P cells remarkedly improved the tube formation ability of HUVECs (Fig. 1H). These findings indicate that PVT1 may exert more important roles in the progression of ccRCC by promoting tumor angiogenesis.

### PVT1 promotes ccRCC proliferation, angiogenesis and metastasis in vivo

**Fig. 2:**
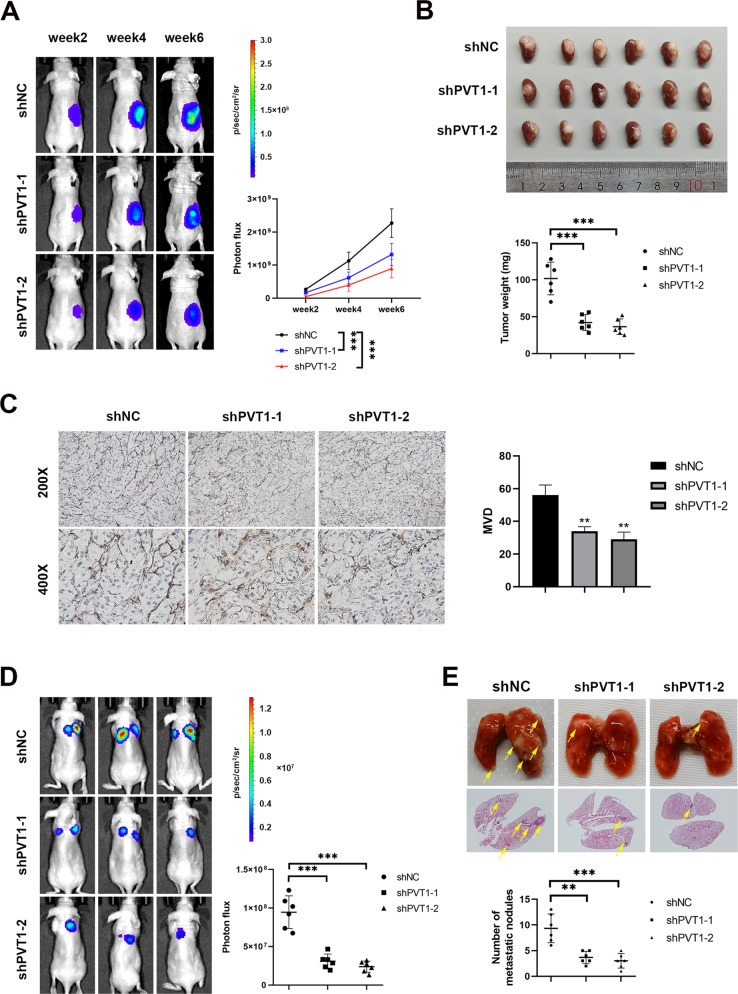
PVT1 promotes ccRCC proliferation, angiogenesis and metastasis in vivo. **A**, **B** Knockdown of PVT1 dramatically inhibited the growth of xenograft in vivo. Representative bioluminescence images of orthotopic tumors performed by a live imaging system (left panel) and statistical analysis (right panel) were shown (**A**). The picture of gross tumors (upper panel) and the final tumor weights (lower panel) were shown (**B**). **C** PVT1-silencing tumors displayed lower microvessel density (MVD). Immunohistochemistry staining with antibody against endothelial cell marker CD34 (left panel) and statistical analysis (right panel) were shown. **P < 0.01, ***P < 0.001. **D** The lung metastasis model showed that PVT1 depletion significantly inhibited ccRCC lung metastasis. Representative bioluminescence images of lung metastases (left panel) and statistical analysis results (right panel) were shown. **E** Nude mice injected with PVT1-silencing 786-O cells had fewer and smaller lung metastases. Representative images of gross and HE staining of the lungs were shown (upper panel). The pulmonary metastatic nodules were counted under a microscope and summarized (lower panel). Arrows indicate pulmonary metastatic nodules. **P < 0.01, ***P < 0.001.

To investigate the potential role of PVT1 in vivo, a renal orthotopic mouse model was employed. PVT1-silencing 786-O cells or counterpart control 786-O cells were injected into the renal subcapsule of BALB/c nude mice. As shown, knockdown of PVT1 dramatically inhibited the growth rate of xenograft in vivo (Fig. 2A, B). Consistently, PVT1-silencing tumors displayed a lower microvessel density (MVD) compared with those of control group (Fig. 2C). In addition, tail-vein injection mouse model was employed, and the results showed that PVT1 depletion dramatically compromised the ability of 786-O cells in promoting lung metastases compared with the control group (Fig. 2D, E). Taken together, our findings indicate that PVT1 may significantly facilitate ccRCC proliferation, angiogenesis and metastasis in vivo.

### PVT1 binds and stabilizes HIF2α in ccRCC cells

**Fig. 3:**
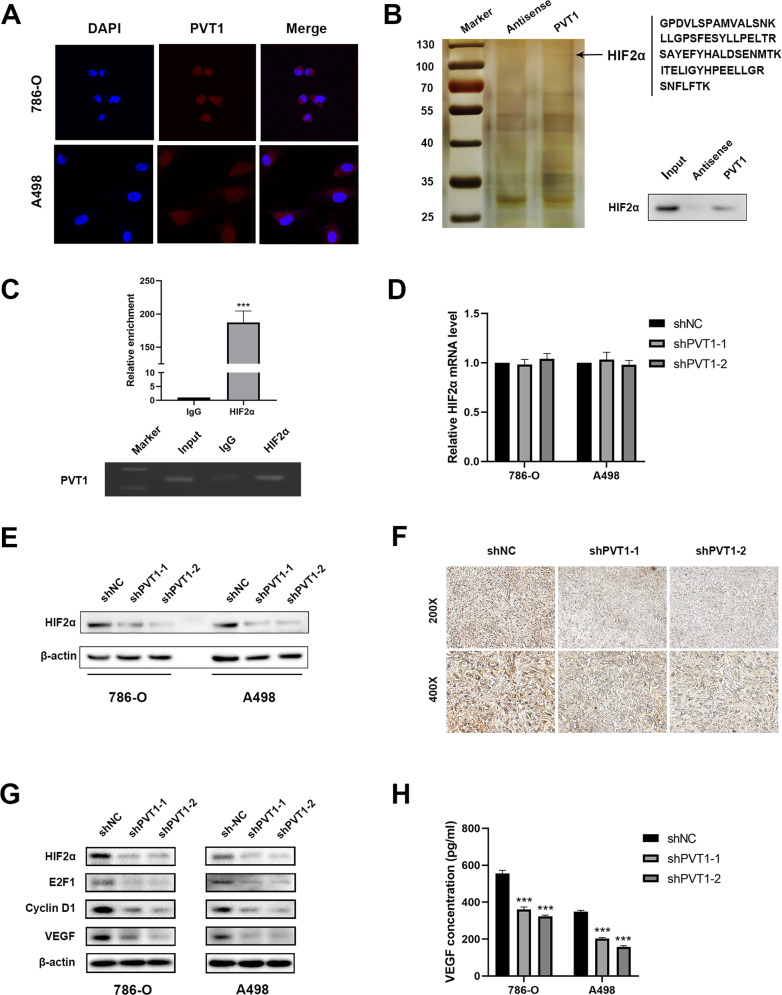
PVT1 interacts with HIF2α and upregulates its expression in ccRCC cells. **A** RNA-FISH showed that PVT1 was predominantly localized in the cytoplasm. Nucleus was labeled with DAPI, and the PVT1 probe was labeled with CY3. **B** HIF2α was identified as the PVT1-interacting protein by RNA pull-down assays. Proteins pulled down by PVT1 or its antisense RNA were separated by SDS-PAGE and subjected to silver staining. A specific band, marked with an arrow, was identified as HIF2α protein in the PVT1 group by LS/MS mass spectrometry (left panel). Western blot showed that HIF2α was pulled down by PVT1 (right panel). **C** RIP assays revealed that PVT1 was remarkably enriched in HIF2α-immunoprecipitated RNAs. IgG was used as a negative control (upper panel). The qPCR products of immunoprecipitated RNAs were analyzed by electrophoresis (lower panel). **D** Knockdown of PVT1 did not affect the mRNA level of HIF2α in ccRCC cells. **E** Knockdown of PVT1 significantly reduced the protein level of HIF2α. **F** IHC assays showed that the protein level of HIF2α was notably decreased in xenografts deriving from PVT1-silencing 786-O cells. **G** PVT1 knockdown significantly reduced protein levels of HIF2α and its downstream targets (E2F1, Cyclin D1 and VEGF) in ccRCC cells. **H** ELISA assays showed that PVT1 knockdown decreased VEGF concentrations in conditioned medium of 786-O and A498 cells. ***P < 0.001.

To point out the underlying molecular mechanism of PVT1 in promoting ccRCC angiogenesis, invasion and migration, we initially performed the RNA-FISH assays and revealed that PVT1 was predominantly located in the cytoplasm rather than the nucleus, indicating that PVT1 may exert its oncogenic effect in the cytoplasm (Fig. 3A). Generally, lncRNAs are prone to function by binding and modulating proteins. Thus, we performed RNA pull-down assays to indentify PVT1-associated proteins (Fig. 3B left panel). Mass spectrometry analysis identified HIF2α as one of the top binding proteins for PVT1 (Supplementary Table 2 and Supplementary Table 3), which was further validated by western blot in independent RNA pull-down assays (Fig. 3B right panel). On the other hand, RIP assays were performed and showed that PVT1 was remarkably enriched in HIF2α-immunoprecipitated RNAs (Fig. 3C). Altogether, these findings indicate that PVT1 is associated with HIF2α in ccRCC cells.

To further dissect the regulatory effect of PVT1 on HIF2α, we measured HIF2α expression in *VHL*-deficient ccRCC cells (786-O and A498) at both RNA and protein levels. The results showed that knockdown of PVT1 did not affect the mRNA level of HIF2α (Fig. 3D), but significantly down-regulated its protein levels in both 786-O and A498 cells (Fig. 3E). In addition, IHC staining of xenografts deriving from PVT1-deficient 786-O cells also displayed lower HIF2α abundance compared with controls (Fig. 3F). Consistently, the expression of well-known HIF2α downstream targets, such as E2F1, Cyclin D1 and VEGF [30–32], were reduced upon PVT1 depletion (Fig. 3G). In addition, PVT1 knockdown significantly decreased the protein production of VEGF in the supernatant derived from 786-O and A498 cells (Fig. 3H).

**Fig. 4:**
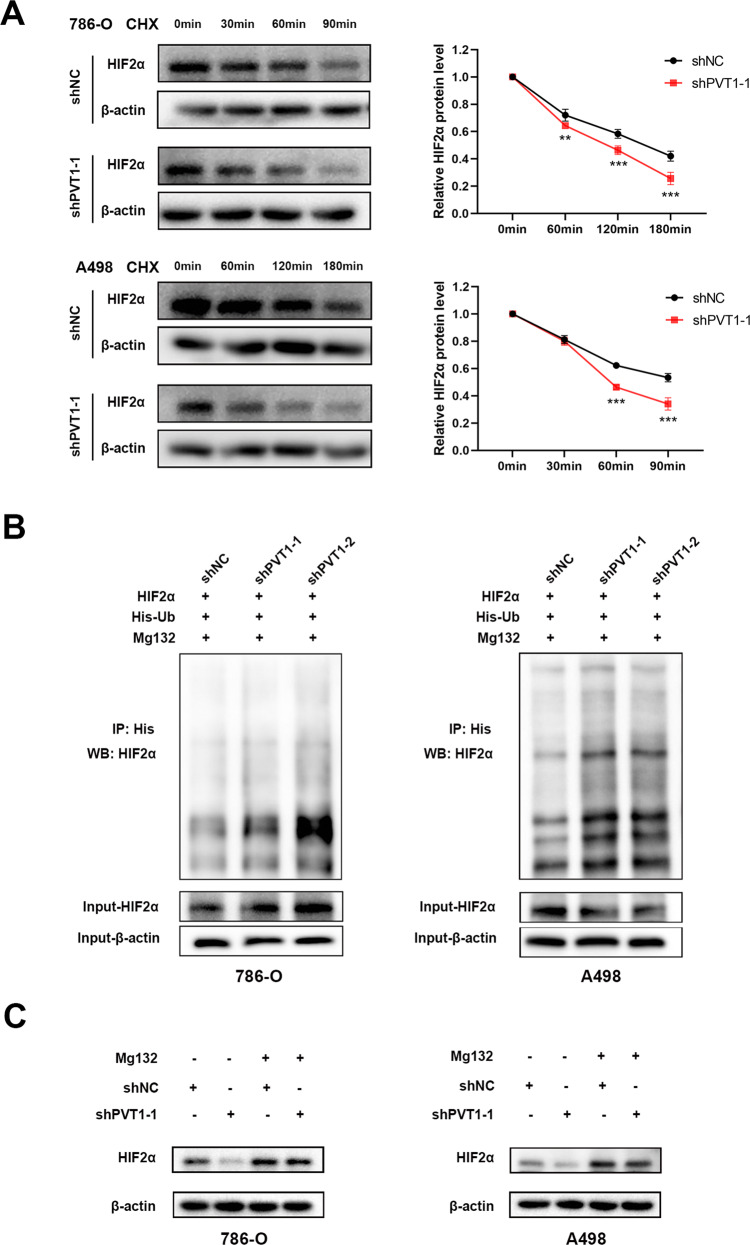
PVT1 protects HIF2α from ubiquitination-mediated degradation. **A** PVT1 knockdown decreased the stability of HIF2α protein. 786-O and A498 cells stably expressing control shRNA or PVT1 shRNA were incubated with cycloheximide (CHX, 100 μg/ml) for the indicated interval, following by western blot (left panel). The quantified level of HIF2α protein was measured by Image J. **B** Ubiquitination assays showed that PVT1 knockdown increased the polyubiquitination level of HIF2α. Cells were transfected with the indicated plasmids for 48 h, and then His-Ub conjugated proteins were pulled down with Ni-NTA agarose beads. The anti-HIF2α antibody was used to detect the polyubiquitination level of HIF2α. **C** The proteasome inhibitor MG132 rescued PVT1 silencing-induced HIF2α reduction. Cells expressing control shRNA or PVT1 shRNA were incubated with MG132 (20 µM) for 4 h before western blot. **P < 0.01, ***P < 0.001.

Since around 70% ccRCC display VHL genetic mutations or deletion, both HIF1α and HIF2α display a relative high level in these tissues. Here we demonstrate that PVT1 could stabilize HIF2α possibly in a post-transcriptional level, thus we tend to further investigate the underlying mechanism. We found that sliencing PVT1 in *VHL*-deficient ccRCC cells could dramatically decreased the stability of HIF2α protein (Fig. 4A), coupled with notably improving HIF2α polyubiquitination (Fig. 4B). Furthermore, PVT1 silencing-mediated down-regulation of HIF2α could be rescued by treatment with proteasome inhibitor MG132 (Fig. 4C). These results together suggest that PVT1 may associate with HIF2α to protect it from ubiquitination-mediated degradation in a pVHL-independent manner.

### PVT1 augments tumor growth and metastasis largely by promoting HIF2α pathway

**Fig. 5:**
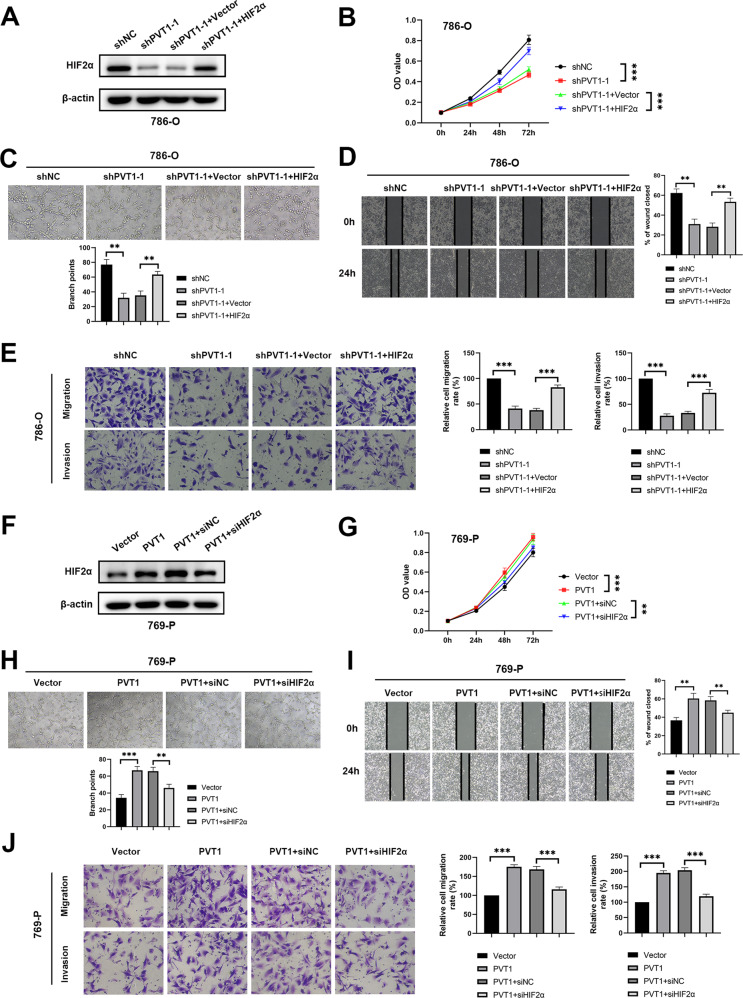
PVT1 accelerates tumor growth and metastasis mainly via promoting HIF2α pathway. **A** Overexpressing HIF2α rescued the PVT1 silencing-induced down-regulation of HIF2α. Overexpressing HIF2α abrogated PVT1 silencing-induced inhibition of proliferation of ccRCC cells (**B**), tube formation of HUVECs (**C**), migration and invasion of ccRCC cells (**D**–**E**). **F** Silencing HIF2α inhibited PVT1 overexpression-mediated up-regulation of HIF2α. Silencing HIF2α abrogated the roles of PVT1 overexpression in promoting proliferation of ccRCC cells (**G**), tube formation of HUVECs (**H**), migration and invasion of ccRCC cells (**I**–**J**). **P < 0.01, ***P < 0.001.

To test whether PVT1 plays its oncogenic role via HIF2α, we overexpressed HIF2α in PVT1-silencing 786-O and A498 cells (Fig. 5A; Supplementary Fig. S2A). As shown, ectopic expression of HIF2α largely abrogated PVT1 silencing-induced inhibition of 786-O and A498 cells proliferation as well as HUVECs tube formation (Fig. 5B, C; Supplementary Fig. S2B, C). Moreover, wound-healing assays, transwell migration and matrigel invasion assays also showed that HIF2α overexpression attenuated the inhibition effects of PVT1 knockdown on ccRCC cells migration and invasion (Fig. 5D, E; Supplementary Fig. S2D, E). Similarly, depletion of HIF2α impaired the promotive effects of PVT1 overexpression on proliferation, migration and invasion of ccRCC cells along with tube formation of HUVECs (Fig. 5F-J). Taken together, our results uncover that PVT1 facilitates ccRCC malignant phenotypes mainly via activating HIF2α pathway.

### HIF2α enhances PVT1 transcription to form a positive feedback regulatory loop

**Fig. 6:**
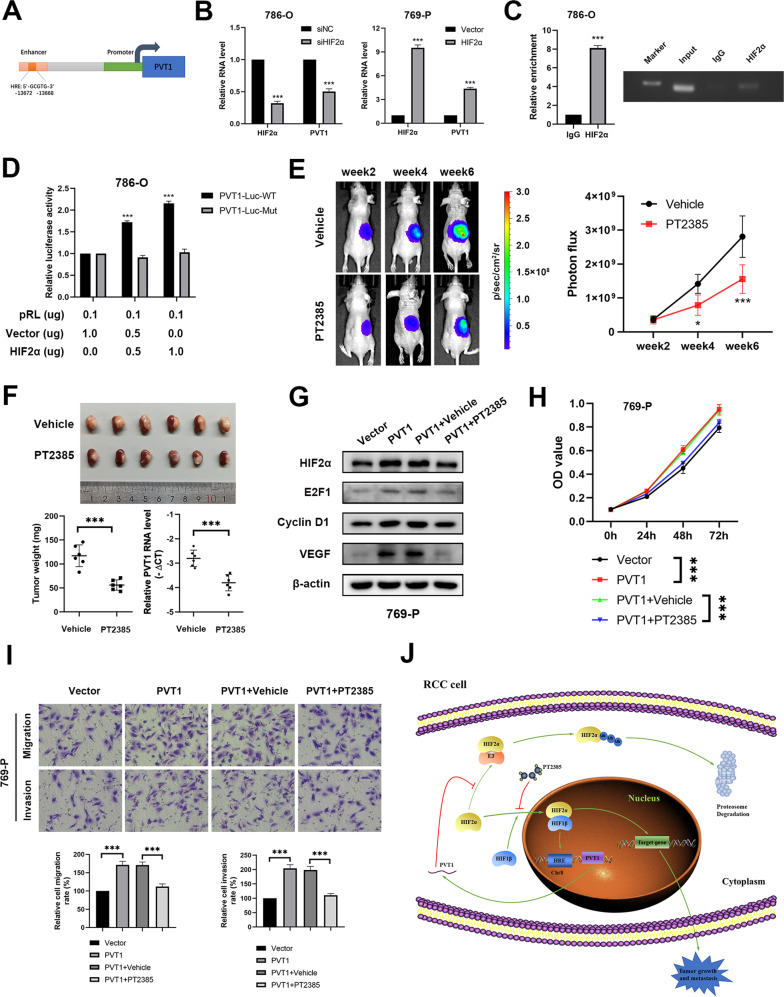
HIF2α enhances PVT1 transcription to form a positive feedback regulatory loop. **A** A schematic diagram of HRE located in the enhancer of PVT1. **B** The RNA level of PVT1 was significantly reduced in HIF2α-silencing 786-O cells and increased in HIF2α-overexpressing 769-P cells. **C** ChIP assays showed that HIF2α bound to the HRE located in the enhancer of PVT1 in 786-O cells. IgG served as a negative control (left panel). The ChIP products were analyzed by electrophoresis (right panel). **D** Dual-luciferase reporter assays showed that overexpression of HIF2α enhanced the activity of luciferase reporter containing wild-type but not mutant PVT1 HRE. **E** PT2385 administration remarkedly inhibited the growth rate of orthotopic tumors. Representative bioluminescence images of orthotopic tumors (left panel) and statistical analysis results (right panel) were shown. **F** The orthotopic xenograft tumor model showed that PT2385 administration significantly inhibited tumor growth in vivo. The picture of gross tumors (upper panel) and the final tumor weights (lower left panel) were represented. qPCR showed that inhibition of HIF2α by PT2385 decreased the expression level of PVT1 in vivo (lower right panel). **G** Western blot showed that PT2385 (20 µM) treatment repressed the upregulation of HIF2α target genes induced by ectopic expression of PVT1. **H-I** PT2385 treatment remarkably attenuated the enhanced cell proliferation, migration and invasion induced by PVT1 overexpression. *P < 0.05, **P < 0.01, ***P < 0.001. **J** A schematic diagram of PVT1/HIF2α positive feedback loop regulating tumor progression in renal cell carcinoma.

It is previously reported that HIF could directly bind to an enhancer located between the MYC and PVT1 gene locus and enhance transcription of these genes [33]. Thus, a hypoxia response element (HRE) has been identified in the PVT1 enhancer, which locates 14 kb upstream of PVT1 (Fig. 6A). In keeping with this finding, we found that the RNA levels of PVT1 were significantly reduced in HIF2α-depleted 786-O cells while increased in HIF2α-overexpressing 769-P cells (Fig. 6B). To further validate the direct regulation of HIF2α on PVT1, ChIP assays were performed and revealed that HIF2α was remarkably enriched within the HRE region of PVT1 (Fig. 6C). On the other hand, we observed that ectopic expression of HIF2α increased the activity of luciferase reporter containing wild-type but not mutant PVT1 HRE (Fig. 6D), indicating that HIF2α positively regulates PVT1 expression, which forms a positive feedback loop, to further enhance HIF2α accumulation and promote tumorigenesis.

### HIF2α inhibitor blocks the PVT1-HIF2α positive feedback loop and its oncogenic roles in ccRCC

As a predominant target of ccRCC, HIF2α has drawn more attention as a result of *VHL* deficiency, and its inhibitors recently have been successfully developed and undergone clinical trial [13–15]. With the evidence that HIF2α transcriptionally regulated PVT1, HIF2α specific inhibitor PT2385 may thus largely compromise PVT1 expression. As expected, in vivo xenograft experiments showed that PT2385 significantly inhibited tumor growth coupled with decreased PVT1 expression (Fig. 6E, F). On the other hand, consistent with our previous finding, the oncogenic roles of PVT1 could be markedly abrogated by PT2385 treatment in vitro (Fig. 6G-I), indicating that PVT1 performs its oncogenic roles at least partially via activating HIF2α pathway. These findings together suggest that HIF2α inhibitor is efficient for combating high-PVT1 or -HIF2α expression ccRCC by blocking the positive feedback regulatory loop between PVT1 and HIF2α (Fig. 6J).

## Discussion

Growing evidence has showed that ubiquitin-dependent protein degradation plays critical roles in post-transcriptional regulation of HIFα. Under normoxia conditions, HIFα is hydroxylated on specific proline residues by prolyl hydroxylases (PHDs) and in turn undergoes proteasomal degradation via a pVHL-dependent pathway [34]. Beside pVHL, other E3 ligases have also been reported to be responsible for the regulation of HIFα via an oxygen-independent pathway. For example, Heat shock protein 90 (Hsp90) was reported to physically interact with HIF1α and enhance its stabilization;[35, 36] receptor of activated protein C kinase (RACK1) competes with Hsp90 for binding to HIF1α and recruits the elongin B & C E3 ligase complex to target HIF1α for proteasomal degradation [37]. Similarly, The E3 ligase cullin 5 also mediates HIF1α degradation in an Hsp90-mediated and oxygen-independent manner [38]. Nevertheless, there is no clear evidence that E3 ligase other than pVHL involves in the degradation of HIF2α. Here we revealed that PVT1 protected HIF2α from polyubiquitination and subsequent proteasomal degradation in *VHL*-deficient renal cancer cells. Thus, exploration of novel E3 ligase interacting with HIF2α and mediating it undergo ubiquitination-proteasome degradation pathway would be an interesting question that should be further investigated in future.

Prior studies have demonstrated that PVT1 is an important oncogene and plays critical roles in onset and development of various cancers [39]. Here we found that PVT1 was dramatically upregulated in ccRCC tissues and predicted shorter survival time, which were consistent with previous studies about the clinical significance of PVT1 in ccRCC [25, 26]. Meanwhile, several studies found that PVT1 exerts oncogenic effects in ccRCC by promoting proliferation, migration and invasion of ccRCC cells [25–27]. In our study, in vitro and in vivo experiments both verified that PVT1 significantly promoted malignant biological behavior of ccRCC cells, especially tumor angiogenesis. PVT1 exerts various biological effects via interacting with different functional proteins, such as increasing the stability of NOP2 [40], or binding with transcriptional factor FOXM1 and STAT3 [19, 23]. Here we identified that PVT1 exerted its angiogenesis role by binding and stabilizing HIF2α in ccRCC cells. More interestingly, PVT1 protects HIF2α from ubiquitination and subsequent degradation in a pVHL-independent manner. This finding further provides evidence that other E3 ligase(s) except pVHL is present to regulate HIF2α stability, thus PVT1 may bind HIF2α to attenuate its binding with E3 ubiquitin ligase or enhance its binding with according deubiquitinase, which is worth to be further investigated.

Although HIF1α and HIF2α undergo a similar regulation by the PHD-mediated hydroxylation and pVHL-mediated ubiqutination, these two proteins also display a distinct function in ccRCC. Among which HIF2α has been considered as an oncogene to promote ccRCC tumorigenesis, while HIF1α exhibits a tumor suppressor role [9–12]. Although PVT1 has been shown to regulate HIF1α transcriptionally by binding with KAT2A and play oncogenic roles in cancers [41], we demonstrated that PVT1 could bind HIF2α and stabilize HIF2α protein, thus promote tumorigenesis and angiogenesis. As a result, the oncogenic functions of PVT1 in ccRCC are mainly mediated by HIF2α but not HIF1α. Thus, HIF2α specific inhibitors could dramatically repress PVT1 oncogenic functions in ccRCC. Reciprocally, HIF2α can increase PVT1 expression by occupying the HRE within the enhancer of PVT1 and form a positive feedback loop, thus the sustained HIF2α will mediate PVT1 oncogenic functions for ccRCC tumorigenesis.

Our study not only reveals a potential mechanism of PVT1 playing oncogenic roles in ccRCC by binding and stabilizing HIF2α, but also points out the positive feedback regulation between lncRNA PVT1 and HIF2α protein, highlighting the promising strategy to combat high-PVT1 expression ccRCC with HIF2α specific inhibitors.

## Materials and methods

### Cell culture and clinical samples

HK-2, 769-P, 786-O, UMRC6, ACHN, RCCJF, A498, CAKI-1 and human umbilical vein endothelial cell (HUVEC) were purchased from the Chinese Academy of Science. HK2, UMRC6, ACHN, A498 and HUVEC were cultured in DMEM medium supplemented with 10% fetal bovine serum (FBS). 769-P, 786-O and RCCJF were grown in RPMI-1640 medium with 10% FBS. CAKI-1 was maintained in McCoy’s 5 A medium with 10% FBS. All cells were cultured in a humidified incubator with 5% CO2 at 37 °C. All cell lines were authenticated by the short tandem repeat DNA profiling test and tested negative for mycoplasma contamination. A total of 140 paired ccRCC samples and matched adjacent normal tissues (ANTs) were collected from Sun Yat-sen University Cancer Center (Guangzhou, China), from December 2007 to December 2018. The clinicopathological information of all 140 patients is provided in Supplementary Table 1. Samples used in this study were approved by Ethical Committee of Sun Yat-sen University Cancer Center (Guangzhou, China). The informed consent of each patient was obtained.

### Plasmid construct, siRNA interference and shRNA transfection

Plasmids encoding HIF2α and ubiquitin were gifts from Professor Jianping Guo (The First Affiliated Hospital of Sun Yat-Sen University, Guangzhou, China). siRNA targeting HIF2α and scrambled siRNA were synthesized by RiboBio (Guangzhou, China). Short hairpin RNA (shRNA) directed against PVT1 and scrambled control were purchased from the GeneChem Company (Shanghai, China). For PVT1 overexpression plasmid, cDNA of PVT1 was synthesized and cloned into GV367 vector (GeneChem, Shanghai, China). 293 T cells were transfected with shPVT1 plasmids and PVT1 overexpression plasmids using Lipofectamine 3000 (Invitrogen, CA, USA), and supernatants containing lentivirus were collected 48 h after the transfection. 786-O, A498 and 769-P cells were transfected with the concentrated lentivirus, followed by incubation with 2 μg/ml puromycin for 2 weeks for selecting the stable transfected cell lines. The siRNA and shRNA sequences were listed in Supplementary Table 4.

### RNA isolation and quantitative real-time PCR (qPCR)

Total RNA was isolated using TRIzol reagent (Invitrogen, CA, USA) according to the manufacturer’s protocol. Briefly, 500 ng of RNA was used for reverse transcription with the PrimeScript™ RT reagent Kit with gDNA Eraser (Takara, Dalian, China). qPCR was performed using SYBR Green Pro Taq HS premix (Accurate Biology, Changsha, China) according to the manufacturer’s protocol. Beta-actin was used as an internal control. The PCR primers were listed in the Supplementary Table 4.

### Western blot

ccRCC cells were lysed with NP-40 lysis buffer according to the manufacturer’s instructions. Then, the protein concentration of each sample was measured using Pierce^TM^ BCA protein assay kit (Thermo, MA, USA). Equal amounts of cell lysates were separated by SDS-polyacrylamide gel electrophoresis (PAGE) and transferred onto a polyvinylidene fluoride (PVDF) membrane. After being blocked in 5% fat-free milk, the PVDF membranes were incubated with primary antibodies overnight at 4 °C. The following antibodies were used: anti-HIF2α antibody (Catalog number: ab199, 1:1000) was purchased from abcam (Cambridge, UK); anti-E2F1 antibody (Catalog number: 3742, 1:1000), anti-Cyclin D1 antibody (Catalog number: 55506, 1:1000) and anti-Beta-actin antibody (Catalog number: 4970, 1:1000) were all purchased from Cell signaling Technology (MA, USA); anti-VEGF antibody (Catalog number: 19003-1-AP, 1:1000) was purchased from Proteintech (Wuhan, China). Then membranes were incubated with HRP-conjugated anti-rabbit IgG at room temperature for 1 h and signal detection was visualized using a western blot substrate kit (Tanon, Shanghai, China).

### MTT and colony formation assays

For MTT assays, a total of 1500 cells were seeded per well in a 96-well plate. MTT assay reagent (Beyotime, Shanghai, China) was added to each well and incubated for 2 h at 37 °C. Newly formed mitochondrial formazan crystals were dissolved by 100 μl DMSO and the absorbance at 490 nm of each well was read on a spectrophotometer. For colony formation assays, a total of 1000 cells were seeded per well in a 6-well plate. After cultured for 2 weeks, the colonies were fixed with 4% paraformaldehyde for 20 min at room temperature and then stained with 0.1% crystal violet. The number of colonies (>50 cells) was counted.

### Tube formation assay

Each well of a 96-well plate was coated with 50 µL matrigel (Corning, NY, USA), and incubated for 30 min at 37 °C. Then HUVECs (20,000 per well) were seeded in the matrigel pre-coated wells and cultured with conditioned culture medium at 37 °C for 6 h. The formation of capillary-like structures was monitored under a light microscope, and counted by Image J software.

### Wound-healing, transwell migration and matrigel invasion assays

For wound-healing assays, the confluent monolayer was scratched using a 200 µL pipette tip. The movement of ccRCC cells was determined in this artificial scratch by monitoring the speed of wound closure. Transwell migration assays and matrigel invasion assays were performed using a 24-well transwell chamber (Corning, NY, USA) with or without matrigel (Corning, NY, USA). About 50,000 cells were resuspended in serum-free medium and seeded onto the upper chamber, and the lower chamber was added with 10% FBS-containing medium as the chemo-attractant. The cells migrated through the membrane or invaded through the matrigel were fixed, stained, and then counted under a light microscope.

### In vivo mouse experiments

All in vivo mouse-related experimental procedures were in compliance with ethical regulations and approved by the Institutional Animal Care and Use Committee of Sun Yat-sen University.

For orthotopic xenograft tumor model, 4-week-old male BALB/c nude mice were used. 18 nude mice were randomly allocated into 3 groups, and anesthetized with 1% pentobarbital (50 mg/kg body weight) by intraperitoneal injection. PVT1-silencing 786-O-luc cells or counterpart control 786-O-luc cells (1×10^6^) were injected into the right renal subcapsule orthotopically. 6 weeks after injection, mice were sacrificed and kidneys with xenograft tumors were removed for further analysis. Orthotopic xenograft weight was estimated by subtracting the weight of the contralateral kidney from the weight of kidney with xenograft tumor.

For drug treatment assays, HIF2α antagonism, PT2385 (AbMole, TX, USA) was suspended in saline containing 2% demethyl sulfoxide (DMSO), 2.5% Tween 80 and 0.5% sodium carboxymethyl cellulose. 12 nude mice bearing 786-O-luc cells orthotopically were randomly divided into 2 groups. 2 weeks after injection of 786-O cells, nude mice of 2 groups were orally treated with vehicle or PT2385 (10 mg/kg body weight) twice daily respectively until the endpoint.

For tail-vein injection lung metastasis assays, 18 nude mice were randomly allocated into 3 groups. PVT1-silencing 786-O-luc cells or counterpart control 786-O-luc cells (1×10^6^) were injected intravenously into each mouse through the tail vein. The experimental mice were sacrificed after 6 weeks. The lung of each mouse was removed and the pulmonary metastatic nodules were counted.

To detect tumor growth and metastasis, nude mice bearing 786-O-luc cells were injected with D-Luciferin (150 mg/kg body weight) intraperitoneally. Bioluminescence signals were collected and analyzed by IVIS Spectrum.

No sample size calculations were performed. Sample size was determined according to our experience as well as literature reporting in terms of specific experiment. Randomization method was used to determine how animals were allocated to different groups. To achieve randomization, all animals were numbered by body weight, then, random number table was used to allocate animals to experimental groups. During the study, no data was excluded from the experiments, and no blinding was done.

### Immunohistochemistry (IHC) staining

Paraffin sections of xenograft tumors were baked in an oven at 65 °C for 1 h. Then, the paraffin sections were deparaffinized in xylene and rehydrated by graded ethanol. After performing antigen retrieval with EDTA antigen retrieval solution, the sections were treated with 3% H_2_O_2_ to block the endogenous peroxidase activity, followed by incubating in blocking buffer (5% bovine serum albumin). Anti-HIF2α antibody (Abcam, catalog number: ab199, 1:100) and anti-CD34 antibody (Abcam, catalog number: ab81289, 1:2500) were added on the sections and incubated in a humidified chamber at 4 °C overnight. Then, the sections were incubated in horseradish peroxidase conjugated goat anti-rabbit IgG (ZSGB-BIO, Beijing, China) for 20 min at room temperature. DAB substrate solution (ZSGB-BIO, Beijing, China) was added on the sections to visualize the staining, followed by counterstaining with hematoxylin, dehydrating and mounting.

### Fluorescence in situ hybridization (FISH) assay

FISH assays were performed with a FISH Kit (GenePharma, Shanghai, China) according to the manufacturer’s protocol. PVT1 probe labeled with CY3 was designed and synthesized by GenePharma Company. Cell nucleus was stained with DAPI. Signals were detected by confocal laser scanning microscopy.

### RNA pull-down and RNA immunoprecipitation (RIP) assays

RNA pull-down was performed using the Pierce™ Magnetic RNA-protein pull-down kit (Thermo Fisher Scientific, MA, USA). Briefly, 3 μg biotin-labeled PVT1 and its antisense transcript were incubated with Streptavidin Magnetic Beads for 30 min at room temperature. Then, 100 μg protein extracting from 786-O cells was added to the RNA-beads mixture, and incubated overnight at 4 °C. The RNA-binding proteins were separated by SDS-PAGE and then visualized by silver staining. Specific protein bands were analyzed by mass spectrometry.

RIP assay was carried out with a Magna RIP^TM^ RNA-binding protein immunoprecipitation kit (Millipore, MA, USA). Briefly, 5 μg of anti-HIF2α and anti-IgG antibodies were incubated with protein A/G beads for 30 min at room temperature respectively. Then 100 μl of 786-O cells lysate was added to the mixture and incubated overnight at 4 °C. After proteinase K digestion, the immunoprecipitated RNAs were extracted, purified, and analyzed by qPCR.

### Enzyme-linked immunosorbent assay (ELISA)

The culture supernatants of ccRCC cells were collected and stored at −80 °C before the test. The concentration of VEGF in supernatant was detected by the Human VEGF ELISA Kit (Abcam, Cambridge, UK) according to the manufacturer’s protocol. The concentration of VEGF was calculated against a standard curve constructed by the absorbances of standard proteins.

### In vitro ubiquitination assay

786-O and A498 cells were transfected with plasmids expressing HIF2α and His-tagged ubiquitin (His-ub). 48 h later, cells were incubated with 20 µM MG132 for 4 h before harvesting. Then, the culture medium was aspirated and 1 ml of PBS was added. Cells were scraped off with a cell scraper and centrifuged at 2500 rpm for 3 min. The cell pellets were resuspended by buffer A (6 M Guanidine-HCl, 0.1 M Na_2_HPO_4_, 0.1 M NaH_2_PO_4_, 10 mM imidazole, pH 8.0). After being sonicated and centrifuged, the cell lysates were incubated with 50 µl Ni-NTA agarose beads for 3 h at room temperature. The pull-down products were washed once with buffer A, once with 1:3 buffer A/buffer TI (25 mM Tris-HCl, 20 mM imidazole, pH 6.8), and twice with buffer TI. The His-Ub conjugated proteins pulled down by Ni-NTA agarose beads were analyzed by western blot as described above.

### Chromatin immunoprecipitation (ChIP) and luciferase reporter assays

ChIP assays were conducted with SimpleChIP Plus Enzymatic Chromatin IP Kit (Cell signaling technology, MA, USA) according to the manufacturer’s instructions. Briefly, after cell culture cross-linking and chromatin digestion, 10 μg of anti-HIF2α and anti-IgG antibodies were incubated with digested chromatin overnight at 4 °C respectively. Then, 30 µl protein G magnetic beads were added in the mixture to incubate for 2 h. The immunoprecipitated DNA was purified and then analyzed by qPCR. The primers used to analyze the enriched DNA are listed in Supplementary Table 4.

For luciferase reporter assays, the PVT1 enhancer with canonical hypoxia response element (HRE) and a mutated version of the HRE sequence were inserted into GV148 vector (GeneChem, Shanghai, China) respectively. ccRCC cells were transfected with HIF2α-expressing plasmid and GV148-based reporter plasmid as described. Meanwhile, CV045 *Renilla* luciferase plasmid (GeneChem, Shanghai, China) was co-transfected as internal control. 48 h after transfection, the cells were lysed and assayed via a dual-luciferase reporter assay kit (Promega, WI, USA). Luciferase activity was normalized to the *Renilla*.

All experiments were replicated for three parallel experiments.

### Statistical analysis

All statistical analyses were performed using R (version 3.6.3). Two-tailed student’s *t*-test was used in the intergroup comparison. The association between clinicopathological parameters and the expression level of PVT1 was analyzed using chi-square test. We calculated the coefficient of variation and comparisons between the groups were performed. *P* value < 0.05 was considered statistically significant.

## Supplementary information

Supplementary information

Supplementary data

Supplementary Table 2

Supplementary Table 3
