# Supplementary material for: Positive feedback regulation of lncRNA PVT1 and HIF2α contributes to clear cell renal cell carcinoma tumorigenesis and metastasis
Source: Oncogene. 2021 Jul 28;40(37):5639–50. doi: 10.1038/s41388-021-01971-7 (PMC8445819; doi:10.1038/s41388-021-01971-7)
Supplement: Supplementary file 1 — Supplementary information [file 41388_2021_1971_MOESM1_ESM.docx]

**Supplementary Fig. S1 PVT1 promotes ccRCC cells proliferation, migration and invasion in vitro.** A Relative PVT1 expression level between RCC and the matched adjacent normal tissues in TCGA KIRC cohort. B Survival analysis showed that high PVT1 expression was correlated with poor OS and PFS in TCGA KIRC cohort. C-F MTT and colony formation assays showed that the proliferation and colony formation were suppressed by PVT1 knockdown (c, e), but promoted by PVT1 overexpression (d, f). G-J The migration and invasion of ccRCC cells were inhibited by knockdown of PVT1 (g, i) and promoted by overexpression of PVT1 (h, j). Numbers of migrated or invaded cells were counted in five random microscopic field and the migration or invasion rate was normalized to the control group. **P < 0.01, ***P < 0.001.

**Supplementary Fig. S2 PVT1 promotes the malignant phenotypes of A498 cells via activating the HIF2α pathway.** A Overexpressing HIF2α rescued the down-regulation of HIF2α induced by PVT1 silencing in A498 cells. B MTT assays showed that simultaneous ectopic expression of HIF2α partially attenuated the proliferation inhibition caused by knockdown of PVT1 in A498 cells. C Tube formation assays showed that ectopically expressed HIF2α partially rescued the inhibition effect of HUVECs capillary tube sprouting induced by the conditioned medium of PVT1-knockdown A498 cells. D-E Wound-healing assays, transwell migration assays and matrigel invasion assays suggested that HIF2α expressing partially rescued the inhibition of migration and invasion abilities of A498 cells induced by knockdown of PVT1. **P < 0.01, ***P < 0.001.

**Supplementary Table 1: Association of PVT1 expression with clinicopathological information in ccRCC.**

**Supplementary Table 2: Proteins pulled down by antisense of PVT1.**

**Supplementary Table 3: Proteins pulled down by PVT1.**

**Supplementary Table 4: Primers, siRNA and shRNA sequences used in this study.**
