# Supplementary material for: Positive feedback regulation of lncRNA PVT1 and HIF2α contributes to clear cell renal cell carcinoma tumorigenesis and metastasis
Source: Oncogene. 2021 Jul 28;40(37):5639–50. doi: 10.1038/s41388-021-01971-7 (PMC8445819; doi:10.1038/s41388-021-01971-7)
Supplement: Supplementary file 2 — Supplementary data [file 41388_2021_1971_MOESM2_ESM.docx]

**Supplementary Fig. S1: PVT1 promotes ccRCC cells proliferation, migration and invasion in vitro.**


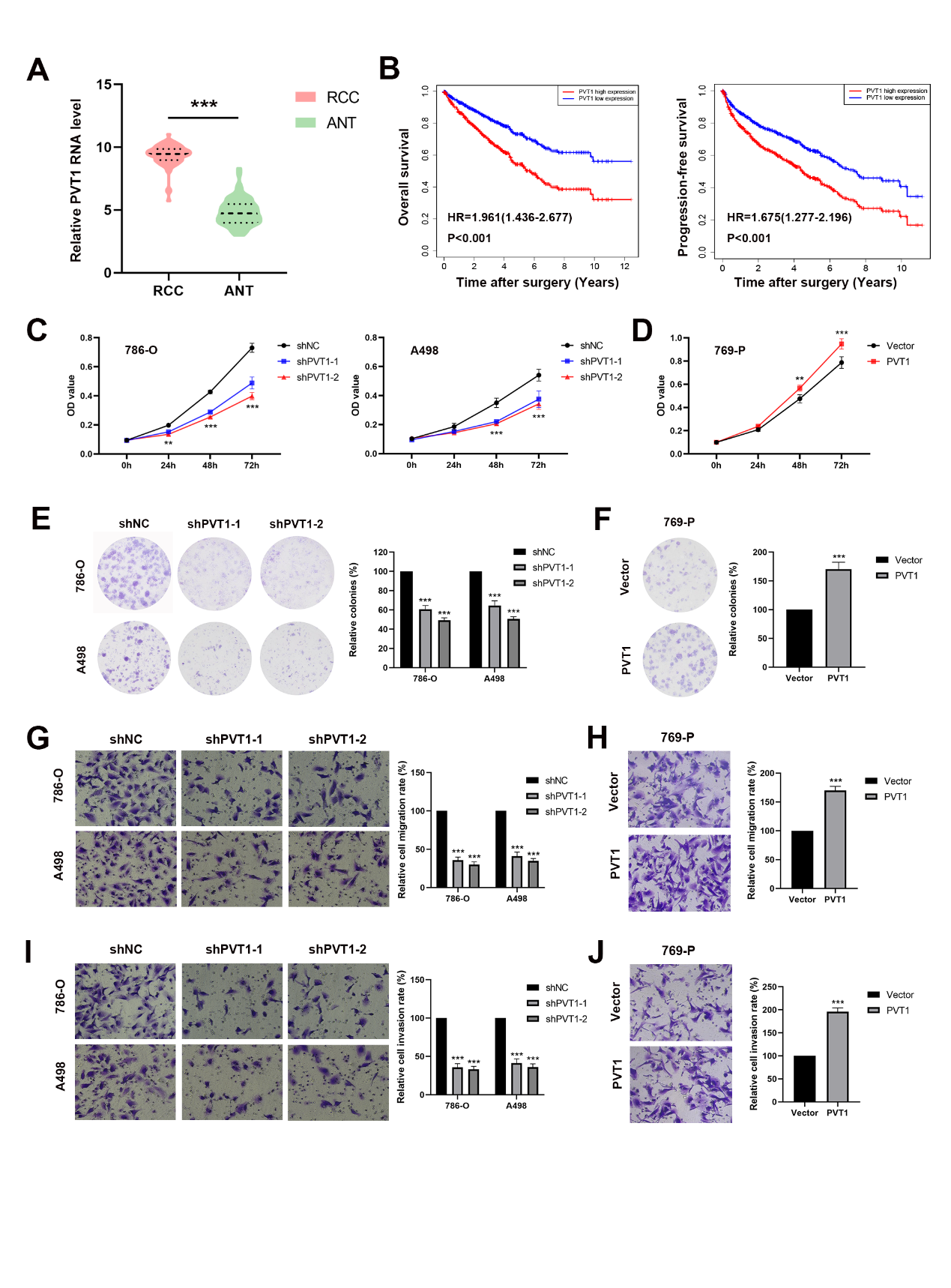


**Supplementary Fig. S2: PVT1 promotes the malignant phenotypes of A498 cells via activating the HIF2α pathway.**


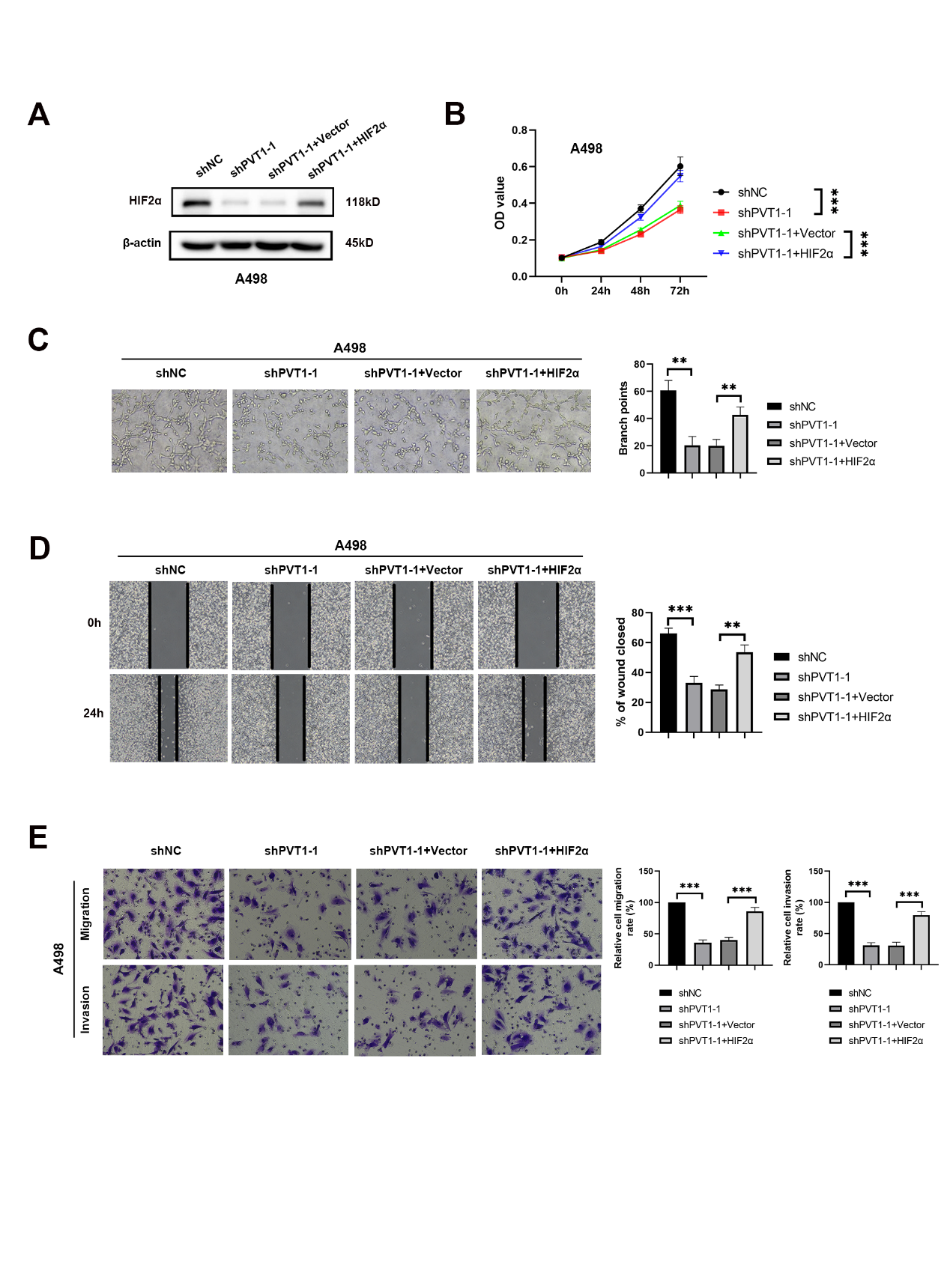


**Supplementary Table 1: Association of PVT1 expression with clinicopathological information in ccRCC.**

| **Variable** | Total（%） | PVT1 expression | | P value |
| --- | --- | --- | --- | --- |
|  |  | Low | High |  |
| **Sex** |  |  |  |  |
| Male | 98 (70.0%) | 45 (32.1%) | 53 (37.9%) | 0.140 |
| Female | 42 (30.0%) | 25 (17.9%) | 17 (12.1%) |  |
| **Age (Median, range)** | 53 (24-80) | 52 (24-79) | 55 (31-80) | 0.054 |
| **T stage** |  |  |  |  |
| 1 | 83 (59.3%) | 47 (33.6%) | 36 (25.7%) | 0.018 |
| 2 | 23 (16.4%) | 12 (8.6%) | 11 (7.8%) | 1/2 VS 3/4 |
| 3 | 27 (19.3%) | 8 (5.7%) | 19 (13.6%) |  |
| 4 | 7 (5.0%) | 3 (2.1%) | 4 (2.9%) |  |
| **N stage** |  |  |  |  |
| 0 | 123 (87.9%) | 66 (47.2%) | 57 (40.7%) | 0.020 |
| 1 | 17 (12.1%) | 4 (2.8%) | 13 (9.3%) |  |
| **M stage** |  |  |  |  |
| 0 | 126 (90.0%) | 67 (47.9%) | 59 (42.1%) | 0.024 |
| 1 | 14 (10.0%) | 3 (2.1%) | 11 (7.9%) |  |
| **AJCC stage** |  |  |  |  |
| I | 78 (55.7%) | 46 (32.9%) | 32 (22.8%) | 0.003  I/II VS III/IV |
| II | 20 (14.3%) | 11 (7.9%) | 9 (6.4%) |  |
| III | 24 (17.1%) | 7 (5.0%) | 17 (12.1%) |  |
| IV | 18 (12.9%) | 6 (4.3%) | 12 (8.6%) |  |
| **Nuclear grade** |  |  |  |  |
| 1 | 26 (18.5%) | 16 (11.4%) | 10 (7.1%) | 0.227 |
| 2 | 82 (58.6%) | 41 (29.3%) | 41 (29.3%) | 1/2 VS 3/4 |
| 3 | 25 (17.9%) | 11 (7.9%) | 14 (10.0%) |  |
| 4 | 7 (5.0%) | 2 (1.4%) | 5 (3.6%) |  |

**Supplementary Table 4: Primers, siRNA and shRNA sequences used in this study.**

| Primers |  |
| --- | --- |
| PVT1-F | TCTGGGGAATAACGCTGGTG |
| PVT1-R | CTTCGTCCCCCATGGACATC |
| ACTB-F | CCTGGCACCCAGCACAAT |
| ACTB-R | GGGCCGGACTCGTCATAC |
| HIF2α-F | ACCTGAAGATTGAAGTGATTGAG |
| HIF2α-R | GTGGCTGGAAGATGTTTGTC |
| ChIP-F | CAGAATCCTTGGGCAAGCTA |
| ChIP-R | GCACCACCTTTGTTCCAACT |
| siRNA (target sequence) |  |
| siHIF2α | GCCGTACTGTCAACCTCAA |
| shRNA (target sequence) |  |
| shPVT1-1 | GCACATTTCAGGATACTAA |
| shPVT1-2 | GCAGCTTATTATAGACTTA |
